# The breakdown of the cytokine network subsequent to human immunodeficiency virus infection

**DOI:** 10.1155/S0962935195000512

**Published:** 1995-09

**Authors:** M. Clerici, M. L. Villa, D. Trabattoni, G. M. Shearer

**Affiliations:** 1 Cattedra di Immunologia Università di Milano Italy; 2 Experimental Immunology Branch National Cancer Institute National Institutes of Health Bethesda MD 20892 USA

## Abstract

The acquired immunodeflciency syndrome (AIDS) is a clinically multifaceted disease induced by infection with the human immunodeficiency virus (HIV). HIV infection results in a complex pattern of immunologic alterations that leads to the development of AIDS in the majority of HIV seropositive (HIV+) individuals. The reduction in CD4 T lymphocyte counts is the hallmark of HIV infection; nevertheless, long before the reduction in CD4 counts reaches critical levels, a series of profound and complex defects that impair the function of CD4 T lymphocytes can be detected. Thus, HIV infection is characterized by quantitative and qualitative defects affecting CD4 T lymphocytes. It was suggested recently that programmed cell death (PCD) is an important mechanism leading to CD4 depletion in HIV infection, and that susceptibility of peripheral lymphocytes to PCD is differentially regulated by diverse cytokines. Thus, type 1 cytokines would protect CD4 lymphocytes against PCD, whereas type 2 cytokines would not protect against, and could augment, PCD. We suggest that the qualitative alterations of the immune response provoke the CD4 depletion characteristic of HIV disease via type 2 cytokinemediated augmentation of PCD, and are therefore ultimately responsible for the progression of HIV infection. Finally, we summarize recent data showing that three correlates of disease progression: emergence of HIV strains with syncitium-inducing ability (SI), type 1-to-type 2 cytokine shift, and CD4 depletion, are significantly associated, suggesting a complex interconnected virologic-immunologic pathogenesis of HIV infection.


Mediators of Inflammation 4, 315–321 (1995)

### The breakdown of the cytokine network subsequent to human immunodeficiency virus infection

M. Clerici,[1,CA] M. L. Villa,[1] D. Trabattoni[1] and G. M. Shearer[2]

[1]Cattedra di Immunologia, Università di Milano, Italy and [2]Experimental Immunology Branch, National Cancer Institute, National Institutes of Health, Bethesda, MD 20892, USA

[CA]Corresponding Author

**THE acquired immunodeficiency syndrome (AIDS) is a clinically multifaceted disease induced by infection with the human immunodeficiency virus (HIV). HIV infection results in a complex pattern of immunologic alterations that leads to the development of AIDS in the majority of HIV seropositive (HIV+) individuals. The reduction in CD4 T lymphocyte counts is the hallmark of HIV infection; nevertheless, long before the reduction in CD4 counts reaches critical levels, a series of profound and complex defects that impair the function of CD4 T lymphocytes can be detected. Thus, HIV infection is characterized by quantitative and qualitative defects affecting CD4 T lymphocytes. It was suggested recently that programmed cell death (PCD) is an important mechanism leading to CD4 depletion in HIV infection, and that susceptibility of peripheral lymphocytes to PCD is differentially regulated by diverse cytokines. Thus, type 1 cytokines would protect CD4 lymphocytes against PCD, whereas type 2 cytokines would not protect against, and could augment, PCD. We suggest that the qualitative alterations of the immune response provoke the CD4 depletion characteristic of HIV disease via type 2 cytokine-mediated augmentation of PCD, and are therefore ultimately responsible for the progression of HIV infection. Finally, we summarize recent data showing that three correlates of disease progression: emergence of HIV strains with syncitium-inducing ability (SI), type 1-to-type 2 cytokine shift, and CD4 depletion, are significantly associated, suggesting a complex interconnected virologic–immunologic pathogenesis of HIV infection.**

**Key words:** Breakdown, Cytokine network, HIV.

### Defective interleukin-2 production as the hallmark of qualitative lymphocyte defects in HIV infection

Early and complex T helper (Th) cells defects have been described in HIV infected individuals.[1–6] We have analysed the qualitative defects of HIV infection by examining *in vitro* antigen- and mitogen-stimulated interleukin (IL)-2 production by PBMC of HIV+ individuals. PBMC were stimulated *in vitro* with recall antigens (influenza virus, tetanus toxoid, or HIV peptides); HLA alloantigens (ALLO); or phytohaemagglutinin (PHA).[5] These stimuli were chosen because they activate different Th-antigen presenting cell (APC) pathways,[7] allowing for a more complete evaluation of the immune system. Thus, recall antigens are exclusively presented by self-APC to autologous CD4; HLA alloantigens can be presented by self-APC to autologous CD4 or they can be directly recognized by autologous CD4 and CD8 T lymphocytes on the surface of allogeneic APC; whereas PHA stimulation of T lymphocytes is only marginally dependent on processation and presentation by APC (see Table 1).[7]

The stimulation of PBMC with this panel of antigens allowed us to recognize that only a minority ($\approx$40%) of HIV+ asymptomatic patients can respond by IL-2 production to recall, ALLO and PHA.[5] Thus, a complex pattern of defects in IL-2 production was observed in the majority of HIV+, asymptomatic individuals. Indeed, approximately 40% of these individuals showed a selective defect in IL-2 production in response to recall antigens, whereas $\approx$10% of them could only respond to PHA, or could not produce IL-2 in response to any stimulation ($\approx$10%).[5] Because all these patients were in the same clinical stage (Walter–Reed stage 1), and

© 1995 Rapid Science Publishers

Table 1. Pathways of T helper cell activation

| Antigen | T helper cell | Antigen presenting cell | IL-2 production |
|---|---|---|---|
| Recall antigens | CD4+ | self APC | ++ |
| HLA alloantigens | CD4+ | self APC | ++ |
| | CD4+ | allo APC | +++ |
| | CD8+ | allo APC | ++ |

PHA-stimulated IL-2 production is marginally dependent on processation and presentation by APC.

had comparable CD4 counts (>400 mmc) the observed functional defects were not secondary to a decrease in CD4 counts.[5] These experiments allowed us to conclude that: (1) defective ability to produce IL-2 can be detected even in the earlier, asymptomatic phases of HIV infection; and (2) these defects are not secondary to either a decrease in CD4 counts or a clinical progression in HIV infection. Similar analyses in vertically transmitted paediatric HIV infection showed that: (1) defective IL-2 production is observed also in the majority of HIV+ paediatric patients;[8] and (2) defective *in vitro* antigen- and mitogen-stimulated IL-2 production is associated in paediatric HIV disease with an augmented incidence of opportunistic and bacterial infections.[8] In the attempt to analyse whether the defective element is the T lymphocyte or the antigen presenting cell, we performed experiments in which cells of HIV-discordant monozygotic twins were matched. The results repeatedly indicated the absence of defects in the ability of HIV+ APC to process and present antigens.[9,10]

We subsequently verified whether defects in IL-2 production could be reverted by treatment with antiretroviral drugs. Thus, we measured *in vitro* stimulated T lymphocyte proliferation and IL-2 production in two cohorts of HIV+ adults and one cohort of paediatric patients, treated with zidovudine, sCD4–IgG or ddI respectively. We observed that all three compounds were capable of temporarily restoring IL-2 production independently of any variation in CD4 counts.[11–13] That this is not an aspecific effect of all antiretroviral drugs was shown by a fourth compound, which was not able to restore T lymphocyte proliferation or IL-2 production (M. Clerici *et al.*, unpublished observations). Additionally, the *in vitro* restoration of IL-2 production by antigen- or mitogen-stimulated peripheral blood mononuclear cells provoked by ddI was associated in paediatric HIV infection with a significant reduction of opportunistic infections during clinical follow-up.[12] Again, the positive changes in clinical parameters was associated with improved T lymphocyte function, but independent of variations in CD4 counts.

### The type 1/type 2 hypothesis of HIV infection

In the late 1980s it was shown by different investigators that murine T helper cells can be functionally distinguished in two subsets which are differentially specialized in furnishing preferential help to cytotoxic T lymphocytes (CTL) or B lymphocytes.[14–17] These two sub-populations, named T helper 1 (TH1) and T helper 2 (TH2), respectively, were subsequently described in humans by Romagnani.[18,19] It was suggested recently that some phenotypic markers may allow us to differentiate TH1 and TH2 lymphocytes. Thus, it was reported that TH2 lymphocytes may express higher quantities of CD30,[20] as well as of a particular isoform of BB-7 (BB-7.2). A third group has observed that CD4+CD7+ T lymphocytes preferentially secrete TH1 cytokines, whereas CD4+CD7− T lymphocytes preferentially produce TH2 cytokines.[21] Nevertheless, no marker has yet been identified that exclusively clusters on TH1 or TH2, and the definition of TH1 and TH2 lymphocytes is still essentially based on the different cytokines that are produced by these cells. Thus, TH1 responses are characterized by secretion of interferon (IFN)-γ and IL-2 and subsequent promotion of cell mediated immunity (CMI), whereas TH2 responses are characterized by secretion of IL-4 and IL-5, with subsequent activation of humoral immunity and generation of antibodies. Interestingly, TH1 and TH2 are cross-regulating as IFN-γ suppresses the activation of TH2 lymphocytes, whereas IL-4 suppresses the production of TH1 cytokines.

IL-2 is a prototypical product of TH1 lymphocytes, and IL-2 production can be down-regulated by TH2 cytokines (classically IL-4). Thus, we began exploring the possibility that the defective IL-2 production observed in HIV+ asymptomatic infection could be accompanied by augmented IL-4 production. We decided to analyse cytokine production by whole PBMC and not by CD4+ T cell clones because we believe cytokine production by whole PBMC to be a more important approximation to the *in vivo* situation.[22,23] In fact, even if T cell clones are an important experimental tool, we were concerned about the artefacts present in the cloning methodology. Our concerns about clones can be summarised as follows: (1) Th cell cloning results in the loss (during the cloning process) of important, non-T accessory cells that produce cytokines with immunoregulatory properties, (2) cloning may

Table 2. TH1 and TH2 cytokines are clonally defined; type 1 and type 2 cytokines are functionally defined

| TH1 cytokines | TH2 cytokines |
|---|---|
| Interferon γ<br>Interleukin-2 | Interleukin-4<br>Interleukin-5 |
| **Type 1 cytokines** | **Type 2 cytokines** |
| Interferon γ<br>Interleukin-2<br>Interleukin-12<br>Interleukin-15 (?) | Interleukin-4<br>Interleukin-5<br>Interleukin-6<br>Interleukin-10<br>Interleukin-13 (?) |

select for populations of T cells, as the techniques involve the selection and expansion of cells in relatively high concentrations of IL-2; and (3) the investigation of PBMC tests for the effects of cytokines produced by multiple cell types on Th cell function, and includes both autocrine and paracrine regulation whereas in cloned T cell tests for the cytokines produced by these clones is limited to autocrine regulation. Because our observations were based on cytokine production by all the cells circulating in the peripheral blood and not on clonal isolation, we decided to identify the cytokine patterns observed as type 1 (mainly CMI-inducing), and type 2 (mainly humoral immunity-inducing).[22,23] Because we defined cytokines on a functional basis, we could enlarge the group of type 1 cytokines to include IL-12 and, probably, IL-15 (all cytokines that mainly stimulate CMI, but are not mainly or exclusively produced by clones of CD4+ T lymphocytes) in addition to IL-2 and IFN-γ. Similarly, IL-6, IL-10 and probably IL-13 were considered to be type 2 cytokines even if they are produced by cells other than CD4+ T lymphocytes, as their main effect is that of stimulating B cell activity and antibody generation (see Table 2).

To measure IL-4, we stimulated PBMC of HIV+ asymptomatic donors *in vitro* with PHA for 48 to 72 h, as suggested by kinetic studies, and we evaluated the amount of IL-4 present in the supernatants using a cell line (a kind gift of Dr William Paul, NIAID, NIH) whose growth is dependent on IL-4. (To simplify the assay, we are now measuring IL-4 using commercially available ELISA kits.) To analyse the production of IL-4 at the molecular level, we also quantified (in collaboration with Dr Thomas Wynn, NIAID, NIH) the expression of IL-4 mRNA in the PBMC of the same patients. We observed that IL-4 production was greatly augmented in PHA-stimulated supernatants of HIV+ individuals as compared to HIV− controls, and that the increased IL-4 production was most likely to be observed in the subset of HIV+ asymptomatic patients showing defective IL-2 generation in response to recall antigens.[24] Similarly, IL-4 mRNA was detected in PHA stimulated PBMC of HIV+ individuals, but not in unstimulated PBMC of the same individuals, or in HIV− controls.[24] Even more important, IL-4 neutralizing antibodies were capable to restore *in vitro* antigen-stimulated proliferation and IL-2 production in the majority of patients secreting high amounts of IL-4.[24]

Because IL-10 was described as able to suppress the secretion of type 1 cytokines and CMI even more powerfully than IL-4, we next decided to measure IL-10 production in HIV+ asymptomatic patients. Similarly to the methods used to generate IL-4, we stimulated PBMC *in vitro* with PHA for 48 h; the amount of IL-10 produced was measured using commercially available ELISA kits, and IL-10 mRNA was quantified using PCR methods. We observed that IL-10 production was augmented in HIV+ asymptomatic patients showing defective IL-2 production, and that the patients with the most severe defective IL-2 production (i.e. inability to generate IL-2 even in response to PHA) generated the highest quantities of IL-10.[25] By analogy to what was observed for neutralizing antibodies to IL-4, *in vitro* antigen-stimulated proliferation and IL-2 production could be restored in the majority of these patients by neutralizing antibodies to IL-10.[25] Similarly, we could restore *in vitro* antigen-stimulated proliferation and IL-2 production, as well as mitogen-stimulated IFN-γ production, by IL-12,[26] a prototypical type-1 cytokine described by Chehimi *et al.* as defective, even in the earlier phases of HIV infection.[27] Thus, we proposed that HIV infection is associated with the progressive impairment of type 1 cytokine production, and the progressive augmentation of type 2 cytokine secretion.[22,23] It is important to underline that these events are observed in the asymptomatic phase of HIV infection, before the development of full blown disease. These data, confirmed by numerous other authors,[28–37] are the experimental basis of the type-1/type-2 hypothesis of HIV infection.

Because cytokine secretion is associated with a quantity of diverse biological effects which are readily measurable, we suggest that the list of these effects, which are frequently observed in HIV+ patients and are presented in Table 3, strongly support the hypothesis of a profound cytokine imbalance in HIV infection. Thus, the impairment of delayed type hypersensitivity reaction is secondary to defective IFN-γ, IL-2 and IL-12 production, whereas hyper-IgE is secondary to the augmented production of IL-4, and hyper-eosinophilia is secondary to the augmented pro-

Table 3. Unfavourable prognostic signs in HIV infection

| |
|---|
| Decreased production of type 1 cytokines (IFN-γ, IL-2, IL-12) |
| Increased production of type 2 cytokines (IL-4, IL-5, IL-6, IL-10) |
| Reduced delayed type hypersensitivity (DTH) reactions |
| IL-4-driven hyper-IgE |
| IL-5-driven hypereosinophilia |

duction of IL-5. All these parameters are predictors of poor prognosis in HIV infection.[38–46] Interestingly, it has recently become evident that defective *in vitro* antigen- and mitogen-stimulated IL-2 production is predictive for subsequent reduction in CD4 counts, the time to acquired immunodeficiency syndrome (AIDS), and time to death.[47]

### Different patterns of disease progression in HIV infection

Higher CD4 counts, better preserved IL-2 secretion, and the absence of the clinical signs of type 1/type 2 cytokine imbalance listed in Table 3 are associated with a lack of progression of HIV infection. It has recently become evident that, although the vast majority of HIV+ individuals progress to AIDS, a minority of HIV+ patients exists that does not develop AIDs or show a critical reduction in CD4 T cells, despite long-lasting infection with HIV.[48] These patients have recently been defined long-term non-progressors (LTNP). Different groups have focused on diverse aspects of this phenomenon, the solution of which holds the possible key to a cure for AIDS. Two biological interpretations can be offered in the attempt to explain long-lasting HIV infection without AIDS. Thus, these patients may have been infected by a defective, less pathogenic HIV variant, or these patients may have a stronger immune response, capable of keeping HIV under control. More likely, a strong immune response is preventing HIV from becoming frankly virulent in these individuals. In support of this hypothesis are the findings by Ho and colleagues, and Fauci and colleagues indicating that strong HIV-specific CTL were observed in two groups of LTNP.[49,50] Additionally, it was recently shown that: (1) preserved T cell function correlates with stable CD4 counts;[51] (2) disease-free survival correlates with *gag*-specific cytotoxic T lymphocyte activity;[52,53] and (3) HIV infected chimpanzees become seropositive but do not progress toward AIDS.[54] Finally, it has recently been verified that neither a type 1-to-type 2 cytokine shift nor programmed cell death are present in HIV+ chimpanzees.[55,56]

We have recently analysed (in collaboration with the Clinica delle Malattie Infettive, Università di Milano) cytokine production in a group of LTNP, and compared the cytokine profile with that observed in a group of patients with progressive HIV infection. As we expected, a strong type 1/weak type 2 cytokine profile was observed in the LTNP, whereas a symmetrically opposite weak type 1/strong type 2 cytokine profile was observed in patients with progressive HIV infection.[57] Interestingly, a significantly increased percentage of CD4+/CD7− T lymphocytes (as remembered above, CD4+/CD7− T lymphocytes predominantly secrete type 2 cytokines) was observed in the individuals with progressive HIV infection, but not in the LTNP.[57]

We have performed (in collaboration with the Cattedra di Pediatria IV, Università di Milano) a second study in a cohort of vertically infected children with different patterns of disease progression. In fact, HIV vertical infection follows a bimodal pattern according to which 20% of vertically infected children develop AIDS and eventually die within the first year of life, whereas the remainder develop AIDS at a constant rate per year, reaching the median at about 5 years after birth.[58] We have thus studied a group of vertically infected HIV+ children who did not develop AIDS within the first year of life, and we identified two subsets of children of comparable age, the first one of which was asymptomatic, while the second one showed severe signs of HIV disease. The results indicated that, despite the presence of severe defects in type 1 cytokine production in both groups of children, a greatly increased type 2 cytokine secretion was characteristic of the symptomatic, but not of the asymptomatic children.[43] Interestingly, a significant association with hyper-IgE (the production of which is stimulated by IL-4 secretion) was observed in the paediatric cohort.[43] Thus, we suggest that a strong type 1/weak type 2 cytokine production is associated with delayed (or absent) progression of HIV infection to AIDS.

### Susceptibility of T lymphocytes to programmed cell death (PCD) is differentially regulated by type 1 and type 2 cytokines

It has been observed that PCD is increased in HIV infection, and it was thus suggested that PCD could be one of the mechanism(s) primarily responsible for CD4 depletion in the progression to AIDS.[59,60] HIV+ lymphocytes undergo PCD in unstimulated conditions, and much more so when stimulated with mitogens.[60,61] One of the major differences between HIV+ and HIV− lymphocytes is that whereas only mitogen-activated HIV− lymphocytes (blasts) will undergo PCD upon a second mitogenic restimulation, even resting (unstimulated) HIV+ PBMC will undergo PCD upon stimulation. Therefore, two serial *in vitro* stimuli are needed to induce PCD in HIV− lymphocytes, whereas a single *in vitro* stimulation will provoke PCD in HIV+ lymphocytes, suggesting that in HIV infection lymphocytes are pre-activated *in vivo* to undergo PCD upon *in vitro* restimulation.[60,61]

Mitogenic stimulation will activate CD4 and CD8 lymphocytes via T cell receptor-mediated stimulation, inducing PCD in both subsets of T lymphocytes. It was recently observed that PCD is differentially regulated by type 1 and type 2 cytokines.[61] Thus, PCD was prevented *in vitro* by IFN-γ, IL-2 and IL-12[61] whereas PCD was not prevented, or was augmented, by IL-4 and IL-10.[61] Even more relevant was the observation that PCD could be prevented by neutralizing antibodies to type 2 cytokines, but could not be prevented, or was even raised by neutralization of type 1 cytokines.[62] We have recently observed that the selective stimulation of CD4+ lymphocytes by recall antigens will induce PCD exclusively in the CD4 subset, a situation that more closely resembles that observed *in vivo*. Even in this situation, PCD was oppositely modulated by type 1 and type 2 cytokines, or by the neutralization of type 1 or type 2 cytokines. Additionally, we verified that PCD is effected by lymphotoxin, and that lymphotoxin is responsible for a soluble-factor mediated amplifying loop which causes PCD in innocent-bystander lymphocytes. Finally, the PCD-inducing effect of lymphotoxin is differentially influenced by type 1 and type 2 cytokines (M. Clerici *et al.*, submitted). We suggest that the impaired production of type 1 cytokines and the augmented generation of type 2 cytokines characteristic of HIV infection results in the destruction of CD4 lymphocytes, which is increased by type 2 cytokines and mediated by lymphotoxin. Thus, antigen stimulation of HIV+ lymphocytes in the presence of abnormally low concentrations of IFN-γ, IL-2 and IL-12, and of abnormally elevated concentrations of IL-4 and IL-10 results in the induction of PCD, instead of the induction of T cell proliferation.

### Virologic and immunologic correlates of poor prognosis are associated

Finally, it was suggested that the development of AIDS is secondary to the emergence of an HIV phenotype with a rapid/high replication rate, a tropism for different cell lines, and the capacity to induce the formation of syncytia *in vitro*.[62–67] To verify whether the isolation of syncytium-inducing HIV is correlated with the type 1-to-type 2 cytokine shift, we have analysed virologic and immunologic parameters in two groups of HIV vertically infected children of comparable age who have or have not progressed to AIDS. We observed that progression to AIDS in paediatric HIV infection is associated with isolation of HIV SI variants and increased production of the type 2 cytokines IL-4 and IL-10. Additionally, we observed that these two parameters are statistically associated and that extensive CD4 loss is associated both with the isolation of SI variants and increased IL-4 production (M. Clerici *et al.*, submitted). These recent data indicate that the virologic and immunologic parameters characteristic of advanced HIV infection are strictly associated, and strongly support a virologic–immunologic pathogenesis leading the appearance of AIDS.

### Conclusions

We suggest that the complex qualitative alterations observed in HIV infection are responsible for the progression of HIV disease to AIDS, and that the dramatic reduction of CD4 T lymphocytes which is the hallmark of this disease is mainly secondary to phenomena of PCD that is increased by a type 1/type 2 cytokine imbalance. Thus, we suggest that every therapeutic approach to HIV infection should consider the necessity to restore the normal functionality of the immune system. These approaches could at least theoretically be based on the utilization of: (1) type 1 cytokines; (2) antibodies neutralizing type 2 cytokines; or (3) pharmacological compounds aimed at the selective stimulation of type 1 cytokine secretion and subsequent augmentation of CMI. It is important to notice that the results of a first clinical trial based on the utilization of IL-2 have shown significant improvement in CD4 counts (possibly via the prevention of IL-2-induced PCD).[68] Thus, we strongly favour approaches based on the restoration of normal cytokine production in the therapy for HIV infection, as we believe that a strong CMI is associated with better prognosis, and will ultimately be more capable of controlling HIV replication and disease progression.

ACKNOWLEDGEMENTS. This work was supported by grants Istituto Superiore di Sanità n. 9204-31 and 9304-40.

### References

1. Lane HC, Depper JM, Greene WC *et al.* Qualitative analysis of immune function in patients with the acquired immunodeficiency syndrome: evidence for a selective defect in soluble antigens recognition. *N Eng J Med* 1985; **313:** 79–84.
2. Smolen JS, Bettleheim P, Koller U *et al.* Deficiency of the autologous mixed lymphocyte reaction in patients with classic hemophilia treated with communical Factor VIII concentrate: correlation with T cell subset distribution, antibodies to lymphadenopathy-associated or human T lymphotropic virus, and analysis of the cellular basis of the deficiency. *J Clin Invest* 1985; **75:** 1828–1834.
3. Giorgi JV, Fahey JL, Smith DC *et al.* Early effects of HIV on CD4 lymphocytes *in vivo*. *J Immunol* 1987; **138:** 3725–3730.
4. Miedema F, Petit AJ, Terpstra FG *et al.* Immunological abnormalities in human immunodeficiency virus (HIV)-infected asymptomatic homosexual men. HIV affects the immune system before CD4+ T helper cell depletion occurs. *J Clin Invest* 1988; **82:** 1908–1916.
5. Clerici M, Stocks NI, Zajac RA *et al.* Detection of three distinct patterns of T helper cell dysfunction in asymptomatic, human immunodeficiency virus-seropositive patients. Independence of CD4+ cell numbers and clinical staging. *J Clin Invest* 1989; **84:** 1892–1899.
6. Shearer GM, Clerici M. Early T helper defects in HIV infection. *AIDS* 1991; **5:** 245–254.
7. Via CS, Tsokos GC, Stocks NI, Clerici M, Shearer GM. Human *in vitro* allogeneic responses: demonstration of three pathways of T helper cell activation. *J Immunol* 1991; **144:** 2524–2528.
8. Roilides E, Clerici M, DePalma L, Rubin M, Pizzo PA, Shearer GM. T helper cell responses in children infected with human immunodeficiency virus type 1. *J Pediatrics* 1991; **118:** 724–730.
9. Clerici M, Stocks NI, Zajac RA, Boswell RN, Shearer GM. Accessory cell function in asymptomatic, human immunodeficiency virus infected patients. *Clin Immunol Immunopathol* 1990; **54:** 168–173.
10. Blauvelt A, Clerici M, Lucey DL *et al.* Functional studies of epidermal Langherans cells and blood monocytes in human immunodeficiency virus-infected individuals. *J Immunol* 1995; **154:** 3506–3515.
11. Clerici M, Landay A, Kessler H, Venzon DV, Lucey DL, Shearer GM. Reconstitution of T helper cell function following zidovudine therapy in HIV infected patients *J Infect Dis* 1992; **166:** 723–730.
12. Clerici M, Roilides E, Butler KM *et al.* Changes in T helper cell function in human immunodeficiency virus infected children during dideoxyinosine therapy as a measure of antiretroviral activity. *Blood* 1992; **80:** 2196–2202.
13. Clerici M, Yarchoan R, Blatt S *et al.* Effect of recombinant CD4-IgG on *in vitro* T helper cell function: data from a phase I/II study on patients with the acquired immunodeficiency syndrome (AIDS). *J Infect Dis* 1993; **168:** 1012–1016.
14. Mosmann TR, Coffman RI. Two types of mouse T helper cell clone: implication for immune regulation. *Immunol Today* 1987; **8:** 223–226.
15. Mosmann TR, Coffman RI. TH1 and TH2 cells: different pattern of lymphokine secretion lead to different functional properties. *Annu Rev Immunol* 1989; **7:** 145–168.
16. Fiorentino DF, Bond MW, Mosmann TR. Two types of mouse T helper cell. IV. Th2 clones secrete a factor that inhibits cytokine production by Th1 clones. *J Exp Med* 1989; **170:** 2081–2089.
17. Swain S. Regulation of the development of distinct subsets of CD4+ T cells. *Res Immunol* 1991; **142:** 14–55.
18. Del Prete GF, De Carli M, Mastromauro C *et al.* Purified protein derivative of *Mycobacterium tuberculosis* and excretory-secretory antigen(s) of *Toxocara canis* expand *in vitro* human T cells with stable and opposite (type 1 helper or type 2 helper) profile of cytokine production. *J Clin Invest* 1991; **88:** 346–350.
19. Romagnani S. Human TH1 and TH2 subsets: doubt no more. *Immunol Today* 1991; **12:** 256–257.
20. Del Prete GF, Maggi E, Pizzolo G, Romagnani S. XD30, Th2 cytokines and HIV infection: a complex and fascinating link. *Immunol Today* 1995; **16:** 76–80.
21. Legac E, Autran B, Merle-Beral H, Katlama C, Debre P. $CD4^{+}CD7^{-}CD57^{+}$ T cells: a new T-lymphocyte subset expanded during human immunodeficiency virus infection. *Blood* 1992; **79:** 1746–1753.
22. Clerici M, Shearer GM. Is HIV associated with a TH1 → TH2 switch? *Immunol Today* 1993; **14:** 107–111.
23. Clerici M, Shearer GM. The TH1/TH2 model of HIV infection: new insights. *Immunol Today* 1994; **15:** 575–581.
24. Clerici M, Hakim FT, Venzon DJ *et al.* Changes in interleukin 2 and interleukin 4 production in asymptomatic, HIV-seropositive individuals. *J Clin Invest* 1993; **91:** 759–765.
25. Clerici M, Wynn TA, Berzofsky JA *et al.* Role of interleukin-10 (IL-10) in T helper cell dysfunction in asymptomatic individuals infected with the human immunodeficiency virus (HIV-1). *J Clin Invest* 1994; **93:** 768–775.
26. Clerici M, Lucey DR, Pinto LA *et al.* Restoration of HIV-specific cell mediated immune responses by interleukin-12 *in vitro*. *Science* 1993; **262:** 1721–1724.
27. Chehimi J, Trinchieri G, Frank I *et al.* IL-12 deficiency in HIV-infected patients. *J Exp Med* 1994; **179:** 1361–1366.
28. Naviskas V, Link J, Warhen B, Persson C, Link H. Increased levels of interferon-gamma (IFN-gamma), IL-4 and transforming growth factor-beta (TGF-beta) mRNA expressing blood mononuclear cells in human HIV infection. *Clin Exp Immunol* 1994; **96:** 59–63.
29. Meeyard L, Otto SA, Keet IPM, Van Lier RAW, Miedema F. Changes in cytokine secretion patterns of CD4+ T cell clones in HIV infection. *Blood* 1994; **84:** 4262–4270.
30. Benjamin D, Knobloch TJ, Dayton MA. Human B-cell interleukin-10: B-cell lines derived from patients with AIDS and Burkitt lymphoma constitutively secrete large quantities of interleukin-10. *Blood* 1992; **80:** 1289–1299.
31. Akridge RE, Oyafuso LKM, Reed SG. IL-10 is induced during HIV-1 infection and is capable of decreasing viral replication in human macrophages. *J Immunol* 1994; **153:** 5782–5788.
32. Emilie D, Fior L, Llorente A *et al.* Cytokines from lymphoid organs of HIV-infected patients: production and role in the immune disequilibrium of the disease and in development of B lymphomas. *Immunol Rev* 1994; **140:** 5–35.
33. Naviskas V, Link J, Warhen B, Persson C, Link H. Increased expression of IL-6, IL-10, TNF-alfa and perforin in blood mononuclear cells in human HIV infection. *AIDS Res Hum Retrovir* (in press).
34. Erard F, Wild MT, Garcia-Sanz JA, LeGros G. Switch of CD8 T cells to noncytokitic CD8–CD4– cells that make Th2 cytokines and help B cells. *Science* 1993; **260:** 1802–1805.
35. Barcellini W, Rizzardi GP, Borghi MO, Fain C, Lazzarin A, Meroni PL. TH1 and TH2 cytokine production by peripheral blood mononuclear cells from HIV-infected patients. *AIDS* 1994; **8:** 757–762.
36. Miedema F, Meyaard L, Koot M *et al.* Changing virus–host interactions in the course of HIV infection. *Immunol Rev* 1994; **140:** 35–72.
37. Maggi E, Giudizi MG, Biagiotti R *et al.* Th2-like CD8+ cells showing B cell helper function and reduced cytolytic activity in HIV-1 infection. *J Exp Med* 1994; **180:** 489–495.
38. Blatt SP, Hendrix CW, Butzin CA *et al.* Delayed type hypersensitivity skin testing predicts progression to AIDS in HIV-infected patients. *Ann Int Med* 1993; **119:** 177–184.
39. Markowitz N, Hansen NI, Wilcosky TC *et al.* Tubercolin and anergy testing in HIV-seropositive and HIV-seronegative persons. *Ann Int Med* 1993; **119:** 185–193.
40. Lucey DR, Zajac RA, Melcher GP and the USAF HIV Study Group. Serum IgE levels in 622 persons with HIV infection: IgE elevation with marked depletion of CD4+ T-cells. *AIDS Res Hum Retrovir* 1990; **6:** 427–429.
41. Israel-Biet D, Labrousse F, Tourani J-M, Sors H, Andrieu JM, Even P. Elevation of IgE in HIV-infected subjects: A marker of poor prognosis. *J Allergy Clin Immunol* 1993; **89:** 68–75.
42. Viganò A, Crupi L, Principi N, Salvaggio A. Elevation of IgE in HIV-infected children and its correlation with the progression of the disease. *J Allergy Clin Immunol* 1995 (in press).
43. Viganò A, Principi N, Villa ML, Trabattoni D, Crupi L, Clerici M. Immunologic characterization of children vertically infected with human immunodeficiency virus with slow or rapid progression. *J Pediatrics* 1995; **126:** 368–374.
44. Fleury-Feith J, Van Nheieu JT, Picard C, Escudier E, Bernaudin JF. Bronchoalveolar lavage eosinophilia associated with pneumocystis carinii pneumonia in AIDS patients. Comparative study with non-AIDS patients. *Chest* 1992; **95:** 1198–1201.
45. Smith KJ, Skelton HG, Drabick JJ, McCarthy WF, Ledsky R, Wagner KF. Hypereosinophilia secondary to immunodysregulation in patients with HIV-1 disease. *Arch Dermatol* 1994; **130:** 119–121.
46. Caterino-de-Araujo A. HIV-1 infection and eosinophilia. *Immunol Today* 1994; **15:** 498–499.
47. Dolan MJ, Blatt SP, Clerici M *et al.* A functional and phenotypic assessment of T-helper cells in HIV-1 infected patients offers independent prognostic information for survival. *J Infect Dis* (in press).
48. Levy JA. HIV pathogenesis and long-term survival. *AIDS* 1993; **7:** 1401–1410.
49. Cao Y, Qin L, Zhang L *et al.* Virologic and immunologic characterization of long-term survivors of HIV type 1 infection. *N Eng J Med* 1995; **332:** 201–208.
50. Pantaleo G, Menzo S, Vaccarezza M *et al.* Studies in subjects with long-term nonprogressive HIV infection. *N Eng J Med* 1995; **332:** 209–216.
51. Keet IPM, Krol A, Klein MR *et al.* Long-term asymptomatic HIV-1 infection with normal and low CD4 counts. *J Infect Dis* 1994; **169:** 1236–1241.
52. Gotch FM, Rowland-Jones S, Nixon DF. Longitudinal studies of HIV gag-specific cytotoxic T lymphocyte responses over time in several patients. In: Racz P, Letvin NL and Gluckman JC eds. *Cytotoxic T Cells in HIV and Other Retroviral Infections.* Karger: Basel 1992; 60.
53. Riviere Y, McChesney M, Porrot F *et al.* Role of HIV specific cytotoxic effector cells in disease progression. XI International Conference on AIDS. Berlin, Germany, 6–11 June 1993 [Abstract WS B 03-4].
54. Nara P, Hatch W, Kessler J, Kelliher J, Carter S. The biology of HIV-1 IIIB infection in the chimpanzee: *in vivo* and *in vitro* correlations. *J Med Primatol* 1989; **18:** 343–355.
55. Heeney J, Jonker R, Koornstra W *et al.* The resistance of HIV-infected chimpanzees to progression to AIDS correlates with absence of HIV-related T cell dysfunction. *J Med Primatol* 1993; **22:** 194–200.
56. Gougeon ML, Garcia S, Heeney J *et al.* Programmed cell death in AIDS-related HIV and SIV infections. *AIDS Res Hum Retrovir* 1993; **9:** 553–560.

57. Clerici M, Meroni L, Balotta C *et al.* Cytokine production and phenotypic analyses of HIV seropositive long term non progressors patients. *J Infec Dis* (in press).
58. Auger I. Incubation periods for paediatric AIDS patients. *Nature* 1988; **336:** 575–577.
59. Gougeon ML, Olivier S, Garcia D *et al.* Mise en evidence d'un processus d'engagement vers la mort cellulaire par apoptose dans les lympocytes de patients infecte par le VIH. *C R Acad Sci Paris. Ser III Sc Vie* 1991; **312:** 529–540.
60. Ameisen JC, Capron A. Cell dysfunction and depletion in AIDS: the programmed cell death hypothesis. *Immunol Today* 1991; **12:** 102–106.
61. Clerici M, Sarin A, Coffman RL *et al.* Type 1/type 2 cytokine modulation of T cell programmed cell death as a model for HIV pathogenesis. *Proc Nat Acad Sci (USA)* 1994; **91:** 11811–11815.
62. Asjo B, Manson-Morferldt L, Albert J *et al.* Replicative capacity of human immunodeficiency virus from patients with varying severity of HIV infection. *Lancet* 1986; **ii:** 660–662.
63. Tersmette M, De Goede REY, Al BJM *et al.* Differential syncytium-inducing capacity of human immunodeficiency virus isolates: frequent detection of syncytium-inducing isolates in patients with acquired immunodeficiency syndrome (AIDS) and AIDS-related complex. *J Virol* 1988; **62:** 2026–2032.
64. Tersmette M, Lange JMA, De Goede REY *et al.* Association between biological properties of human immunodeficiency virus variants and risk for AIDS and AIDS mortality. *Lancet* 1989; **i:** 983–985.
65. Fenyo EV, Albert J, Asjo B. Replicative capacity, cytopathic effect and cell tropism of HIV. *AIDS* 1989; **3** (suppl. S-1): 5–12.
66. Schuitemaker, H *et al.* Biological phenotype of human immunodeficiency virus type 1 clones at different stages of infection: progression of disease is associated with a shift from monocytotropic to T-cell-tropic virus populations. *J Virol* 1992; **66:** 1354–1360.
67. Koot M *et al.* Prognostic value of HIV-1 syncytium-inducing phenotype for rate of CD4+ cell depletion and progression to AIDS. *Ann Intern Med* 1993; **118:** 681–688.
68. Anonymous. Immune based therapies for HIV infection. Meeting report. *Clinical Immunology Spectrum* 1993; **6:** 18–21.

**Received 26 May 1995; accepted 29 May 1995**
